# Evaluation and clinical significance of the stomach age model for evaluating aging of the stomach-a multicenter study in China

**DOI:** 10.1186/1472-6890-14-29

**Published:** 2014-06-28

**Authors:** Qin-Yan Gao, Zhen-Hua Wang, Yun Cui, Jian-Qiu Sheng, Kun-He Zhang, Rui-Hua Shi, Jian-Ming Xu, Wei-Chang Chen, Xiu-Li Zuo, Shu-De Li, Yue-Xiang Chen, Yan-Yan Song, Jing-Yuan Fang

**Affiliations:** 1 GI Division, Renji Hospital, Shanghai Jiao-Tong University School of Medicine, Shanghai Institution of Digestive Disease, 145 Middle Shandong Rd, Shanghai 200001, China; 2 Key Laboratory of Gastroenterology & Hepatology, Ministry of Health, Shanghai Jiao-Tong University, 145 Middle Shandong Rd, Shanghai 200001, China; 3 State Key Laboratory of Oncogene and Related Genes, 145 Middle Shandong Rd, Shanghai 200001, China; 4 The Military General Hospital of Beijing PLA, 5 East sishitiao nanmencang, Beijing 100700, China; 5 The First Affiliated Hospital of NanChang University, 17 Yongwaizheng Street, Nanchang 330006, China; 6 The First Affiliated Hospital of NanJing Medical University, 300 Guangzhou Rd, Nanjing 210029, China; 7 The First Affiliated Hospital of Anhui University, 218 Jixi Rd, Hefei 230022, China; 8 The First Affiliated Hospital of SooChow University, 188 Shizi Street Suzhou 215006, China; 9 Qilu Hospital of Shandong University, 107 West Wenhua Rd, Jinan 250012 China; 10 Second Military Medical University Changhai Hospital, 168 Changhai Rd, Shanghai 200433, China; 11 Second Military Medical University Changzheng Hospital, 415 Fengyang Rd, Shanghai 200003, China; 12 Department of Statistics, Shanghai Jiao-Tong University School of Medicine, 227 South Chongqing Rd, Shanghai 200025, China

**Keywords:** Stomach Age, Chronic atrophic gastritis, Gastric cancer surveillance, Model

## Abstract

**Background:**

A higher prevalence of chronic atrophic gastritis (CAG) occurs in younger adults in Asia. We used Stomach Age to examine the different mechanisms of CAG between younger adults and elderly individuals, and established a simple model of cancer risk that can be applied to CAG surveillance.

**Methods:**

Stomach Age was determined by FISH examination of telomere length in stomach biopsies. Δψm was also determined by flow cytometry. Sixty volunteers were used to confirm the linear relationship between telomere length and age while 120 subjects were used to build a mathematical model by a multivariate analysis. Overall, 146 subjects were used to evaluate the validity of the model, and 1,007 subjects were used to evaluate the relationship between prognosis and Δage (calculated from the mathematical model). ROC curves were used to evaluate the relationship between prognosis and Δage and to determine the cut-off point for Δage.

**Results:**

We established that a tight linear relationship between the telomere length and the age. The telomere length was obvious different between patients with and without CAG even in the same age. Δψm decreased in individuals whose Stomach Age was greater than real age, especially in younger adults. A mathematical model of Stomach Age (real age + Δage) was successfully constructed which was easy to apply in clinical work. A higher Δage was correlated with a worse outcome. The criterion of Δage >3.11 should be considered as the cut-off to select the subgroup of patients who require endoscopic surveillance.

**Conclusion:**

Variation in Stomach Age between individuals of the same biological age was confirmed. Attention should be paid to those with a greater Stomach Age, especially in younger adults. The Δage in the Simple Model can be used as a criterion to select CAG patients for gastric cancer surveillance.

## Background

Chronic atrophic gastritis (CAG) was listed as precancerous condition for gastric cancer by the WHO in 1978. It has been observed that a moderately increased risk of gastric cancer with the presence of CAG after 10 years of follow-up [1]. Because CAG tends to be lifelong, and spontaneous healing is rare [2], it is no doubt that younger adults with CAG would have a higher risk of gastric cancer due to the long duration of disease.

Some experts believe that the pathological degenerative changes of CAG may be a semi-physiological phenomenon especially in the elderly population [3]. However, a high prevalence among younger adults is found in some high-risk regions for gastric cancer in Asia [4]. Our research seeks to investigate the different underlying causes of CAG between different age groups.

Telomere erosion can be regarded as a biological clock [5]. As telomeres shorten with age in humans and premature aging syndromes are often associated with shortened telomeres, it has been proposed that telomere length is an important parameter in aging. Telomere length reduction has been confirmed in diseases such as hypertension [6], diabetes and coronary heart disease [7,8], and it has been also confirmed in cells of the human thyroid and parathyroid [9], as well as liver, kidney and skin [10]. However, whether this is also the case with CAG or in the stomach aging process remains unclear.

To address these questions, the quantification of telomere length is essential. Recent studies have described a method of fluorescence *in situ* hybridization (FISH) that is faster and more reliable than comparable techniques [11-14], which can also be applied to paraffin-embedded tissue samples [15]. Using FISH, we were able to measure the telomere length of chromosomes in stomach cells from biopsy samples obtained by endoscopy.

In this study, we define a new term of “Stomach Age”, which can be used to evaluate the aging of the stomach and is based on the telomere length of stomach cells. We attempted to use this concept to explain the phenomenon of CAG in younger adults and to establish a simple mathematical model to calculate Stomach Age, which can be applied clinically for prognostication.

## Methods

### Study subjects and archived tissue samples to establish the Stomach Age model

Tissue samples were obtained from the pathology archives of the GI Division at Shanghai Jiao-Tong University School of Medicine, Renji Hospital and eight other medical centers (Military General Hospital of Beijing PLA, First Affiliated Hospital of Nanchang University, First Affiliated Hospital of Nanjing Medical University, First Affiliated Hospital of Anhui University, First Affiliated Hospital of Soo-Chow University, Qilu Hospital of Shandong University, Second Military Medical University, Changhai Hospital and Changzheng Hospital). Formalin-fixed paraffin-embedded blocks were retrieved from 246 subjects (60 healthy volunteers and 186 out-patients at gastrointestinal clinical departments) who underwent endoscopy. At least two biopsies were taken from the antrum and corpus on both the greater curvature and the lesser curvature of the stomach, and then routinely embedded in paraffin blocks, sectioned and stained. Tissue sections (3–4 μm) from each sample were used for the histopathological examination and FISH. Another two biopsies taken from the antrum were preserved in liquid nitrogen and kept at -80°C for flow cytometry.

### Study population to evaluate the predictive ability of the Stomach Age model

Relevant information from 1,200 patients at the Shanghai Jiao-Tong University School of Medicine, Renji Hospital was analyzed. At the baseline timepoint, all participants underwent endoscopic examination with biopsies, which were repeated at least once at subsequent visits during the follow-up period. The relevant information regarding the histological results and the answers to the questionnaire, described below, were recorded and compared between age groups.

### Questionnaire

We interviewed all participants using a standard self-administered questionnaire to assess baseline characteristics. The mean time for completing the questionnaire was approximately 15–20 minutes. The questionnaire included the following: 1) demographic factors; 2) any family history of medical conditions, including cancer; 3) a detailed medication history; 4) sleeping habits and quality, and job pressures; 5) the number of cigarettes smoked per day and the duration of smoking; 6) the frequency and amount of water, milk, beverages, wine and hard liquor taken; 7) tea and coffee drinking habits; 8) the consumption of rice, meat, fish, vegetables, fruits, pickled vegetables or salted fermented products, and dehydrated foods; and 9) favorite flavors, cooking methods, and intake of night snacks.

### Histopathological diagnosis and ***Helicobacter pylori*** infection assessment

Each stained slide was examined independently by two experienced pathologists who were unaware of the individual’s clinical details using the updated Sydney classification [16]. When there were disagreements, the biopsies were re-examined until agreement was reached. Two histopathological entities were defined: chronic non-atrophic gastritis (CNAG) and CAG. CNAG was defined as any grade of inflammation with no atrophy in both the corpus and the antrum. CAG was defined as atrophy or/and intestinal metaplasia (IM) in either the corpus or the antrum. The histological features of the biopsies were recorded as slight, moderate, or severe according to the Sydney scoring system. Patients with no or inadequate biopsies were excluded from the study. *Helicobacter pylori* infection was detected by either rapid urease testing of biopsy samples or [13]^13-^C urease breath test.

### FISH

Hybridization was performed according to the manufacturer’s instructions. Briefly, slides were deparaffinized and rehydrated through a series of ethanol gradients, treated with 0.1% Tween 20 detergent in de-ionized water, incubated in citrate buffer (Target Unmasking Solution; Vector Laboratories, Burlingame, USA) in steam, immersed in de-ionized water and fixed in ethanol gradients, and air-dried. Approximately 10 μl of PNA probe was added to each slide. The PNA probe for telomeric sequences was a ready-to-use probe included in the Telomere PNA Fish Kit/FITC (K5327; DakoCytomation A/S, Glostrup. Denmark). The slides were then coverslipped, incubated at 84°C for 5 min, and hybridized for 2 h at room temperature in the dark. After extensive washing, first at 65°C and then at room temperature with PBST. The nuclei were then stained with DAPI (1 μg/ml, Molecular Probes, Eugene, OR), after washed in stilled water, the slides were mounted with antifade mounting media (Prolong™ Anti-fade Mounting Media; Molecular Probes, Eugene, OR, USA).

### Microscopy and data analysis

Slides were imaged using an epifluorescence microscope (Nikon Eclipse 80i). The fluorescence excitation/emission filters used were FITC 490 nm BP excitation via a XF38 filter set. Fluorescence images were captured with a cooled charge-coupled device camera (Nikon DSRil). For the tissue section, the calculated areas were identified according to the H&E-stained slides before the fluorescence microscopy which contained the epithelial cells and the glands (pyloric glands in antrum and fundic glands in corpus). After that the calculated areas were divided into five regions automatically by the professional software (Image-Pro Plus 6.0). At least 1000 nuclei were calculated in each region. Five regions of each tissue section were examined for telomere labeling; the intensity of the fluorescence was utilized to reflect this. Telomere signals were quantitated by a method validated recently in which the sum of pixel intensities in the FITC channel for a given cell nucleus is normalized to the DAPI signal. DAPI staining provides a robust measure of DNA content, being largely insensitive to cell type, proliferation status, and degree of chromatin condensation [17-19]. Each region of the tissue section was examined for telomere labeling independently by two experienced technicians at different time. The correlation coefficients were estimated.

### Flow cytometry and measurement of Δψm

Δψm, the voltage across the inner mitochondrial membrane, can be altered by oxidative damage, such as that which occurs during the aging process. Studies showed that Δψm decreased gradually with age [20,21]. So we believed that Δψm can be used to evaluate the aging of the stomach and as a measurement to validate the term of “Stomach Age”. To support our study, we determined the value of Δψm of those patients. Antrum biopsy samples were first prepared as single cell suspensions using a tissue dissociation kit according to the manufacturer’s protocol (KGA829, KeyGEN Biology Co., Ltd, Nanjing, China). For staining, the cells were incubated with JC-1 (Molecular Probes, Invitrogen, Germany) for 30 min at 37°C in the dark. The cells were then washed in PBS and analyzed immediately by flow cytometry (FACScan; BD Biosciences, Franklin Lakes, NJ, USA). A total of 10,000 cells were examined for green fluorescence using a 529 nm filter and for orange fluorescence using a 590 nm filter. All data were analyzed using BD Cell Quest Pro Software (BD Biosciences).

### Statistical analysis

Statistical analysis was performed using SPSS 17.0 statistical software. The paired *t*-test, Chi-square test and analysis of variance were used. All data were presented as the mean ± SE. Differences were considered to be statistically significant at *P* < 0.05. Multivariate analysis was performed by logistic regression using a backward elimination procedure for variables not significantly associated with the Δage (Stomach Age-real age; *P* = 0.05) [22]. The sensitivity and specificity of the models were calculated. The performance of the predictive model was assessed by the area under the receiver operating characteristic curve (AUROC). We chose the highest Youden’s index as the optimal cut-off point [23].

### Ethics

The study protocol was accepted by the Renji Hospital Ethics Committee (Shanghai, China). Each participant was required to sign an informed consent form.

## Results

### Linear relationship between telomere length and age in stomach cells

**Figure 1:**
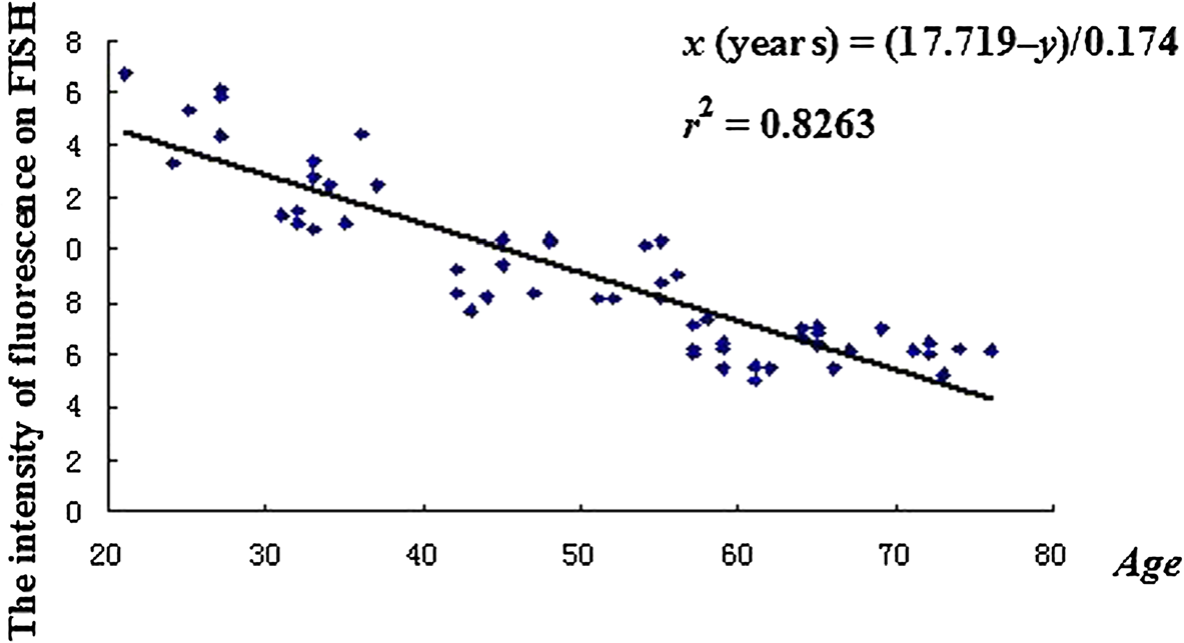
**Stomach Age of volunteers.** The volunteers’ age and the intensity of fluorescence on fluorescence *in situ* hybridization are closely correlated in a linear manner (*r*^2^ = 0.8263) when a simple regression analysis is applied (*y* = -0.174*x* + 17.719, where *y* is fluorescence intensity and *x* is Stomach Age). Rearranging this equation, the Stomach Age model is *x* (years) = (17.719 –*y*)/0.174.

In order to evaluate the Stomach Age, we aimed to establish the standard criteria of Stomach Age in healthy people. Sixty antrum biopsies from healthy volunteers were used to establish the standard criteria. These included 30 males and 30 females aged 23–76 years, in whom endoscopy showed a generally normal gastric lining and in whom the Stomach Age was considered to be the same as their real age. Five regions of each tissue section were examined for the intensity of telomere labeling; the average intensity was used for the regression equation. The mean coefficient of variation was 0.097 which we consider satisfactory. The volunteers’ characteristics and fluorescence intensity are listed in Table 1. As demonstrated in Figure 1, a tight linear relationship (goodness of fit: *r*^2^ = 0.8263) existed between the intensity of telomere labeling and the age. The equation of the computed regression line is *x* (years) = (17.719–*y*)/0.174. In this equation, *y* was the value of fluorescence intensity on FISH and *x* was the age. The correlation coefficient between FISH data and age is r = √0.8263 = 0.91. Thus, we clearly demonstrated a linear decline in telomere length with age increasing. By using the equation above the biological aging level of stomach (Stomach Age) of every one could be expressed precisely in terms of the intensity of fluorescence on FISH and compared individually.

**Table 1:** The characteristics and fluorescence intensity of volunteers

**Age range (years)**	**21-30**	**31-40**	**41-50**	**51-60**	**61-70**	**71-80**
Total number	6	12	9	15	12	6
Number of male	3	9	6	9	6	3
Fluorescence intensity (mean ± SD)	14.8 ± 0.69	11.8 ± 0.92	9 ± 1.07	7.87 ± 1.64	6.18 ± 0.76	6.15 ± 0.04
Occupation						
Student	3	0	0	0	0	0
Professional	0	4	4	2	1	0
Administrative	0	2	1	3	1	0
Sales	1	3	1	0	0	0
Blue-collar	1	2	1	0	0	0
Freelance	1	1	2	0	0	0
Retired	0	0	0	10	10	6
Family history						
Stomach cancer	0	0	0	0	0	0
Other cancer	0	0	1	0	0	0
History of peptic ulcer	0	0	0	0	0	0
Smoking status & history	0	2	2	1	1	0
Seldom	0	1	0	0	1	0
<10 cigarettes per day	0	1	2	1	0	0
10-20 cigarettes per day	0	0	0	0	0	0
>20 cigarettes per day	0	0	0	0	0	0
Less than 5 years	0	1	0	0	0	0
5-10 years	0	1	1	0	0	0
More than 10 years	0	0	1	1	1	0
Alcohol drinking habits	0	2	1	2	1	0
<250 ml per day	0	2	1	2	0	0
>250 ml per day	0	0	0	0	0	0
Less than 5 years	0	1	0	0	0	0
More than 5 years	0	1	1	2	1	0
Coffee habits	2	1	0	0	0	0
Tea drinking habits	1	2	2	1	0	1

### Different telomere lengths between CAG and CNAG patients

**Figure 2:**
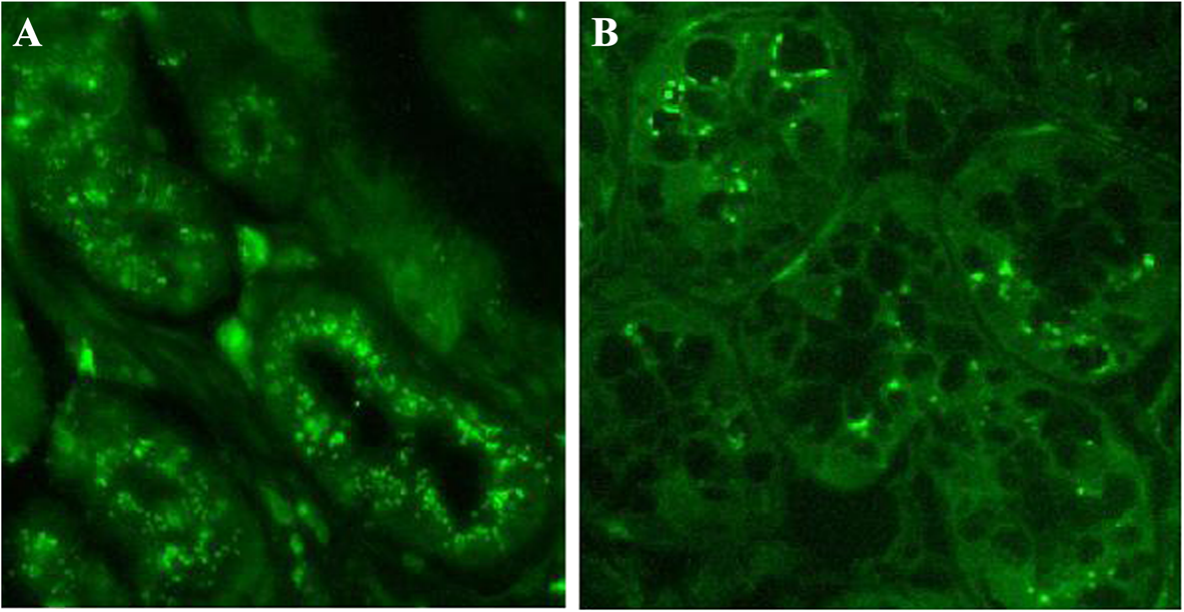
**Fluorescence *in situ *hybridization (FISH) of stomach biopsies.** FISH was performed on biopsy samples obtained from patients by endoscopy. Telomeres are stained with a FITC-labeled antitelomeric probe and are colored green. Picture **A** (male, 40 years old, endoscopy result: CNAG) demonstrates strong telomeric signals in normal stomach. Picture **B** (male, 40 years old, endoscopy result: CAG with moderate intestinal metaplasia) demonstrates weak telomeric signals in atrophic tissue, indicating that CAG may accelerate aging of the stomach. Original magnification: ×40 **(A, B)**.

Secondly, we found that telomere length was obvious different between patients with and without CAG. At the same age, the intensity of the fluorescence of was lower in CAG patients than CNAG patients (Figure 2), which suggests that CAG may accelerate stomach aging. This change can make the degree of stomach aging in young patients with CAG equivalent to that of elderly individuals. Therefore, we postulated that people of the same biological age may have a different Stomach Age, which could be influenced by risk factors such as gastric atrophy and IM.

### Flow cytometry and measurement of Δψm

To obtain further evidence to support our hypothesis, we determined the value of Δψm. If CAG do accelerate stomach aging, the Δψm of those patients should decrease simultaneously.

JC-1(5′,6,6’-tetrachloro-1,1′3,3’-tetraethylbenzimidazolylcarbocyanine iodide) can be used as an indicator of mitochondrial potential, which, when damaged, plays a central role in the aging process [24]. In cells that have mitochondria with a high Δψm, JC-1 forms orange fluorescent J-aggregates (Q2 region), whereas in cells with depolarized or damaged mitochondria, the sensor dye is present as green fluorescent monomers (Q4 region). We calculated the telomere length of enrolled subjects (146 subjects), and then obtained the Stomach Age of those subjects by using the Stomach Age equation (obtained from the health volunteers). After that, we compared their Δψm values.

**Figure 3:**
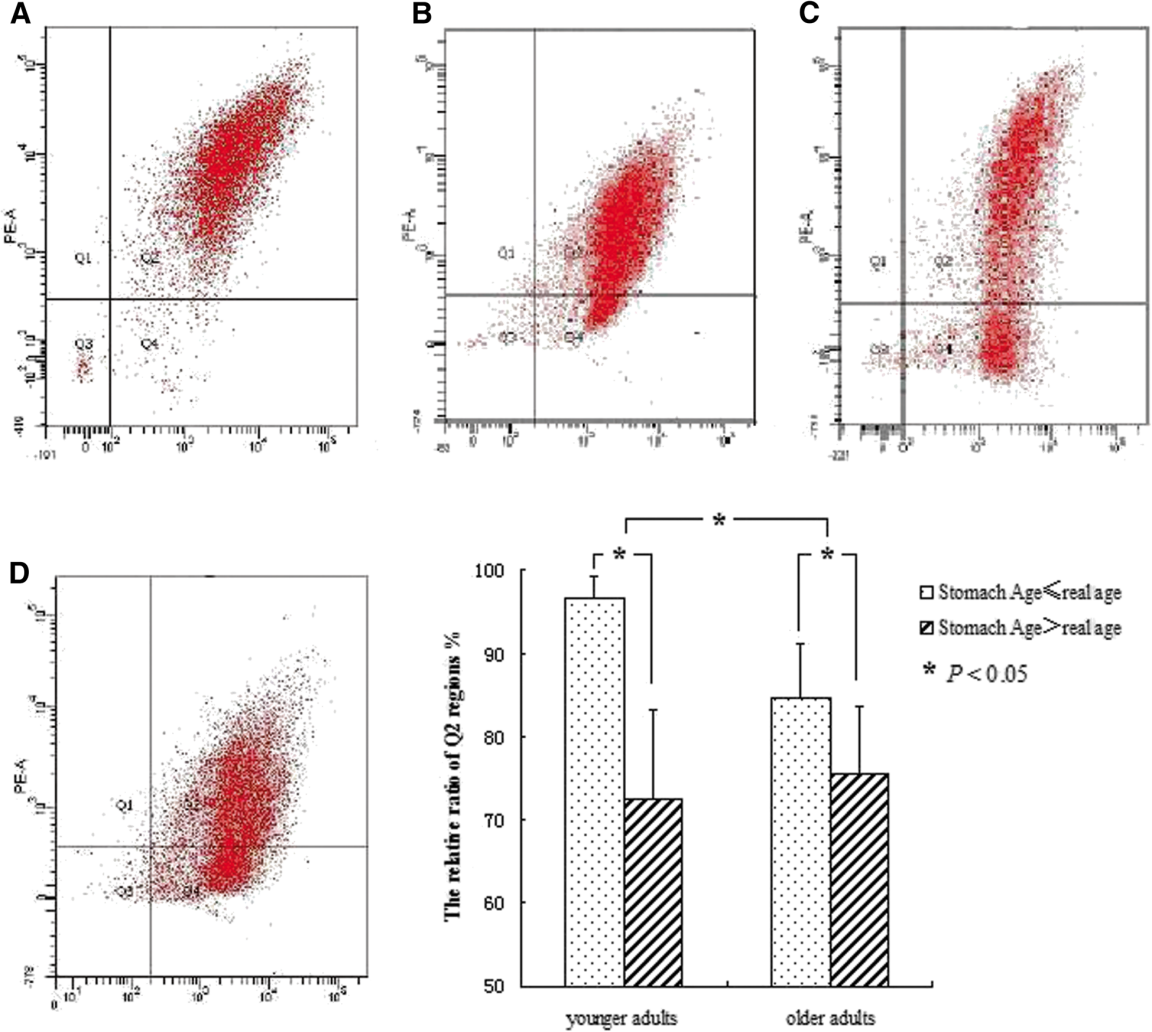
**Measurement of mitochondrial membrane potential (Δψm). A)** Overall, 90.82% of cells in the Q2 region and only 2.03% in Q4 (male, 32 years old, Stomach Age 31 years, endoscopy result: CNAG); **B)** 83.91% of cells in Q2 and 13.49% in Q4 (female, 77 years old, Stomach Age 76 years, endoscopy result: CNAG); **C)** 69.06% of cells in Q2 and 25.92% in Q4 (female, 30 years old, Stomach Age 52 years, endoscopy result: CAG with slight atrophy and intestinal metaplasia); **D)** 65.29% of cells in Q2 and 31.37% in Q4 (female, 65 years old, Stomach Age 74 years, endoscopy result: CAG with slight atrophy and moderate intestinal metaplasia). The difference is significant within each group and between the two groups (*P* < 0.05).

We defined individuals below 50 years of age as the younger adult group, and individuals aged ≥50 years were the older adult group (Figure 3). In CNAG patients of younger adults, the Stomach Ages were younger than or equal to their real age, most of the sample cells were localized in the Q2 region (high Δψm). However, in CAG patients of younger adults, the Stomach Ages were greater than their real age, most cellular dots shifted from Q2 to the Q4 region, indicating a loss of Δψm, and the relative ratio of cellular dots in the Q2 regions decreased significantly (*P* < 0.05). The same trend occurred in the older adults, but the shift in ratio was not as great as in the younger group. These results indicate that CAG could accelerate stomach aging accompany with the decreasing of Δψm. The Δψm was decreased in individuals whose Stomach Age was greater than their real age, especially in younger adults (*P* < 0.05). Stomach Age can represent the biological age of the stomach more precisely than the true biological age.

### Evaluation and establishment of the Simple Model in a multicenter study

For use in clinical practice, we tried to modify the equation of the Stomach Age to obtain a Simple Model. 120 samples including 60 volunteers were used (The detailed characteristics are listed in Table 2). Firstly, we measured the samples’ intensity on FISH, and then calculated each individual’s Stomach Age using the equation.

**Table 2:** The baseline variables and histological evaluation of 120 subjects for the establish model

	**Number of male**						**Number of female**					
	**(30 out-patients/30 healthy volunteers)**						**(30 out-patients/30 healthy volunteers)**					
Age range	**<30**	**30-39**	**40-49**	**50-59**	**60-69**	**>69**	**<30**	**30-39**	**40-49**	**50-59**	**60-69**	**>69**
Number	5	12	14	10	14	5	5	11	17	11	12	4
H.*pylori* infection positive	1	2	1	2	0	0	1	2	3	2	1	0
Histopathologic results												
Atrophy	2	3	8	1	8	2	2	8	14	5	6	1
Intestinal metaplasia	0	1	4	1	6	1	1	4	10	3	4	1
Mild Dysplasia	0	0	0	1	1	1	0	0	1	0	0	0
Occupation												
Students	2	0	0	0	0	0	3	0	0	0	0	0
Professional	0	2	2	1	1	0	0	5	4	1	0	0
Administrative	0	1	1	2	1	0	0	1	5	1	0	0
Sales	1	2	1	1	3	0	1	1	2	0	0	0
Blue-collar	1	3	5	1	0	0	0	1	1	0	0	0
Freelance	1	4	5	0	2	0	1	3	5	2	7	0
Retired	0	0	0	5	7	5	0	0	0	7	5	4
High job pressure	3	3	4	3	3	0	1	5	8	1	2	0
Sleeping time												
>8 h	1	5	4	2	3	0	1	4	3	5	1	0
6-8 h	3	2	5	2	3	5	3	2	7	5	5	2
<6 h	1	5	5	6	8	0	1	5	7	1	6	2
Poor quality of sleeping	1	4	1	1	2	1	1	2	0	2	1	1
Family history												
Stomach cancer	1	0	0	1	0	0	1	0	0	0	0	1
Other cancer	1	0	1	0	0	0	0	0	0	1	0	0
History of peptic ulcer	1	0	1	0	0	0	1	0	2	1	0	0
Smoking status & history												
Seldom	0	1	0	1	1	0	0	1	0	0	0	0
<10 cigarettes per day	0	2	2	1	2	1	0	0	0	0	0	0
10-20 cigarettes per day	0	0	0	0	0	0	0	0	0	0	0	0
>20 cigarettes per day	0	0	0	0	0	0	0	0	1	0	0	0
Less than 5 years	0	2	0	0	0	0	0	1	0	0	0	0
5-10 years	0	1	1	1	0	0	0	0	0	0	0	0
More than 10 years	0	0	1	1	3	1	0	0	1	0	0	0
Coffee habits	0	1	1	0	0	0	2	2	1	2	1	0
Less than 5 years	0	1	1	0	0	0	2	1	0	1	1	0
More than 5 years	0	0	0	0	0	0	0	1	1	1	0	0
Tea drinking habits												
Green tea	2	4	3	4	2	2	0	3	0	1	0	0
Black tea	0	1	1	0	0	0	0	0	1	2	0	0
Others	0	0	0	2	0	0	0	0	1	0	0	0
Less than 5 years	1	4	0	3	0	0	0	1	0	0	0	0
More than 5 years	1	1	4	3	2	2	0	2	2	3	0	0
Alcohol drinking habits	0	4	3	2	1	0	0	0	0	0	0	0
<250 ml per day	0	2	3	2	0	0	0	0	0	0	0	0
>250 ml per day	0	2	0	0	0	0	0	0	0	0	0	0
Less than 5 years	0	3	0	0	0	0	0	0	0	0	0	0
More than 5 years	0	1	3	2	1	0	0	0	0	0	0	0
Spicy flavors	1	2	2	1	0	0	1	3	3	2	1	1
Regular meals	5	5	9	6	8	4	1	5	7	6	10	4
Lengths of meals												
<10 minutes	4	4	5	2	1	0	3	2	5	4	3	0
10-30 minutes	0	8	8	3	10	2	2	3	10	5	8	1
>30 minutes	1	0	1	5	3	3	0	6	2	2	1	3
Drinking liquid at eating time	2	5	8	6	5	2	5	3	10	2	3	2
Consumption of milk	2	7	7	5	4	2	3	9	8	5	6	2
Consumption >2 per week												
Salted fermented products	0	5	3	4	7	2	1	5	5	6	4	1
Desserts	2	2	3	0	3	0	4	2	4	3	1	0
Dehydrated foods	2	3	3	0	1	0	2	4	2	1	0	0
Fried foods	1	6	2	2	1	0	3	4	8	4	4	0
Leftovers	2	6	6	3	10	5	0	4	11	9	5	1
Seafood	1	7	7	1	2	0	3	5	3	2	3	1
Pickled vegetables	0	1	0	2	3	2	0	2	4	6	2	2

We also collected their baseline variables from a questionnaire and the histological information, and several risk factors and Δage (The age calculated from the FISH –real age) were analyzed by multivariate regression analysis using SPSS 17.0. After backward selection, several factors remained in the final model, as shown in Table 3, the Simple Model was found to be:

**Table 3:** Multivariate logistic regression model for simple Stomach Age

**Variable**	**Parameter estimate**	**Standard error**	**F value**	**Pr > F**
Intercept	1.8704	0.4832	16.68	<.0001
Pathological result of endoscopy				
X1 inflammation	-0.44783	0.25975	11.27	0.0010
X2 activity	-3.39746	1.84974	6.19	0.0139
X3 atrophy	1.71968	0.76847	8.81	0.0035
X4 intestinal metaplasia (none 0, mild 1, moderate 2, severe 3)	3.15415	1.59460	15.99	0.0001
X5 family history of gastric cancer (yes 1, no 0)	13.99844	5.82543	5.77	0.0202
X6 accompany with cardiovascular disease (yes 1, no 0)	18.62093	5.95565	9.78	0.0030
X7 accompany with Gastro-esophageal reflux disease (yes 1, no 0)	-16.95696	4.90988	13.09	0.0004
X8 times of fast and junk food per week	18.16387	3.57430	10.55	0.0014

$$\begin{array}{l} \Delta \mathrm{age}\;=\;1.87–0.45*X1–3.39*X2\;+\;1.72*X3\\ \;+\;3.15*X4\;+\;13.99*X5\;+\;18.62\\ \;*X6–16.96*X7\;+\;18.16*X8, \end{array}$$

and

$$\mathrm{Stomach}\;\mathrm{Age}\left(\mathrm{years}\right)\;=\;\mathrm{real}\;\mathrm{age}\;+\;\Delta \mathrm{age}.$$

The Hosmer–Lemeshow goodness-of-fit test suggested that the model was well calibrated (*P* > 0.05).

**Figure 4:**
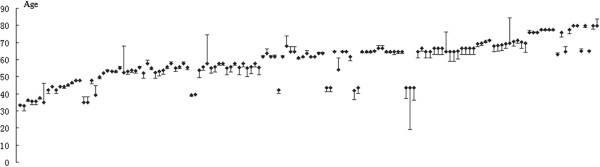
**No significant difference was found between the Simple Model and the FISH data (n = 146).** For each subject, Stomach Age was determined using both the samples’ intensity on FISH and the Simple Models. Each dot represents a subject. The error bars represent the difference between the biological age which calculated from the samples’ intensity on FISH and the Simple Models of Stomach Age. Almost all of the subjects showed a short error bars which indicates that the Simple Model can be used in clinical practice instead of the FISH. The paired *t*-test was used to compare the two results, and no significant difference was found (*P* > 0.05).

One-hundred and forty-six subjects from multiple centers were used to evaluate the accuracy of the Simple Model. For each subject, Stomach Age was determined using both the samples’ intensity on FISH and the Simple Models. The paired *t*-test was used to compare the two results, and no significant difference was found (*P* > 0.05, detailed materials were shown in Figure 4), which indicates that the Simple Model can be used in clinical practice instead of the FISH.

### The relationship between Δage and prognosis in a large sample study

Overall, 1,200 patients were recruited in the study, subjects were asked to reply to the standard questionnaire, which encompassed the eight factors in the Simple Model of Stomach Age, by telephone or letter. A total of 1,007 subjects, 457 male and 560 female, returned completed questionnaires. The average duration of follow-up was 5.4 years (range, 12 months to 190 months).

We calculated the Stomach Age of each subject by the Simple Model, and the Δage were recorded. The histological results at the beginning and the end of the follow-up period were compared individually. On the basis of the comparison of histological results, 1,007 subjects were divided into three groups: Group A (329 subjects), whose histological results had improved after follow-up as the degree of inflammation or/and atrophy or/and IM was reduced after; Group B (320 subjects), in whom there was no change after follow-up; and Group C (358 subjects), whose biopsy results worsened, meaning the degree of inflammation or/and atrophy or/and IM had increased, and newly emerging atrophy or/and IM was present.

**Figure 5:**
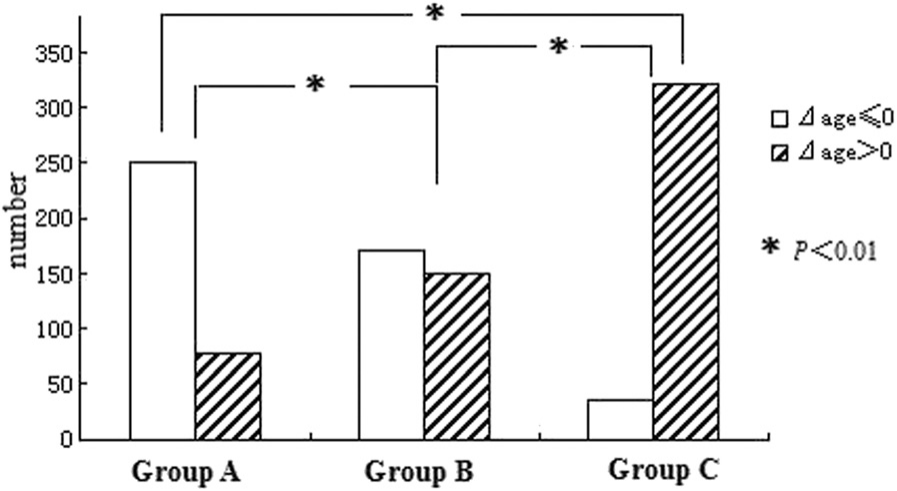
**The comparison of Δage in groups with a different disease prognosis.** In Group A (CAG improved), the majority of subjects were Δage ≤0 (Stomach Age of these patients will be younger than their real age) on the contrary, the majority of subjects in Group C (worsened CAG) were Δage >0 (Stomach Age greater than their real age). And no significant difference in the subjects composition of Stomach Age in Group B (no change). In multiple comparisons, there was a significant difference between the three groups (*P* < 0.01), and the significant difference also existed between each group (*P* < 0.01).

We found that in Group A, the majority of subjects (75.99%) were Δage ≤ 0 (Stomach Age was younger than or equal to their real age), and on the contrary, the majority of subjects in Group C (88.82%) were Δage > 0 (Stomach Age was greater than their real age). In multiple comparisons, there was a significant difference between these three groups (*P* < 0.01; Figure 5).

After this, we divided the 1,007 subjects into subgroups according to their real age in order to observe whether there was a significant impact on prognosis according to Stomach Age. The results showed that Stomach Age could influence the prognosis of disease at all biological ages analyzed (Table 4).

**Table 4:** The comparasions between groups of different age

**Age**		**20-39**		**40-49**		**50-59**		**60-69**		**70-79**		**≥80**	
		N^*^	***P***^***#***^	**N**	***P***	**N**	***P***	**N**	***P***	**N**	***P***	**N**	***P***
GroupA	Δage ≤ 0	8		25		75		79		53		12	
	Δage > 0	2		6		27		29		7		6	
GroupB	Δage ≤ 0	4	0.046	24	0.000	64	0.000	54	0.000	18	0.000	6	0.000
	Δage > 0	8		16		50		52		17		7	
GroupC	Δage ≤ 0	2		7		7		11		8		1	
	Δage > 0	4		24		67		126		68		33	

^*^N = number of patients.

^*#*^*P* = *P* value.

### Evaluate the predictive ability of the Simple Model of Stomach Age

**Figure 6:**
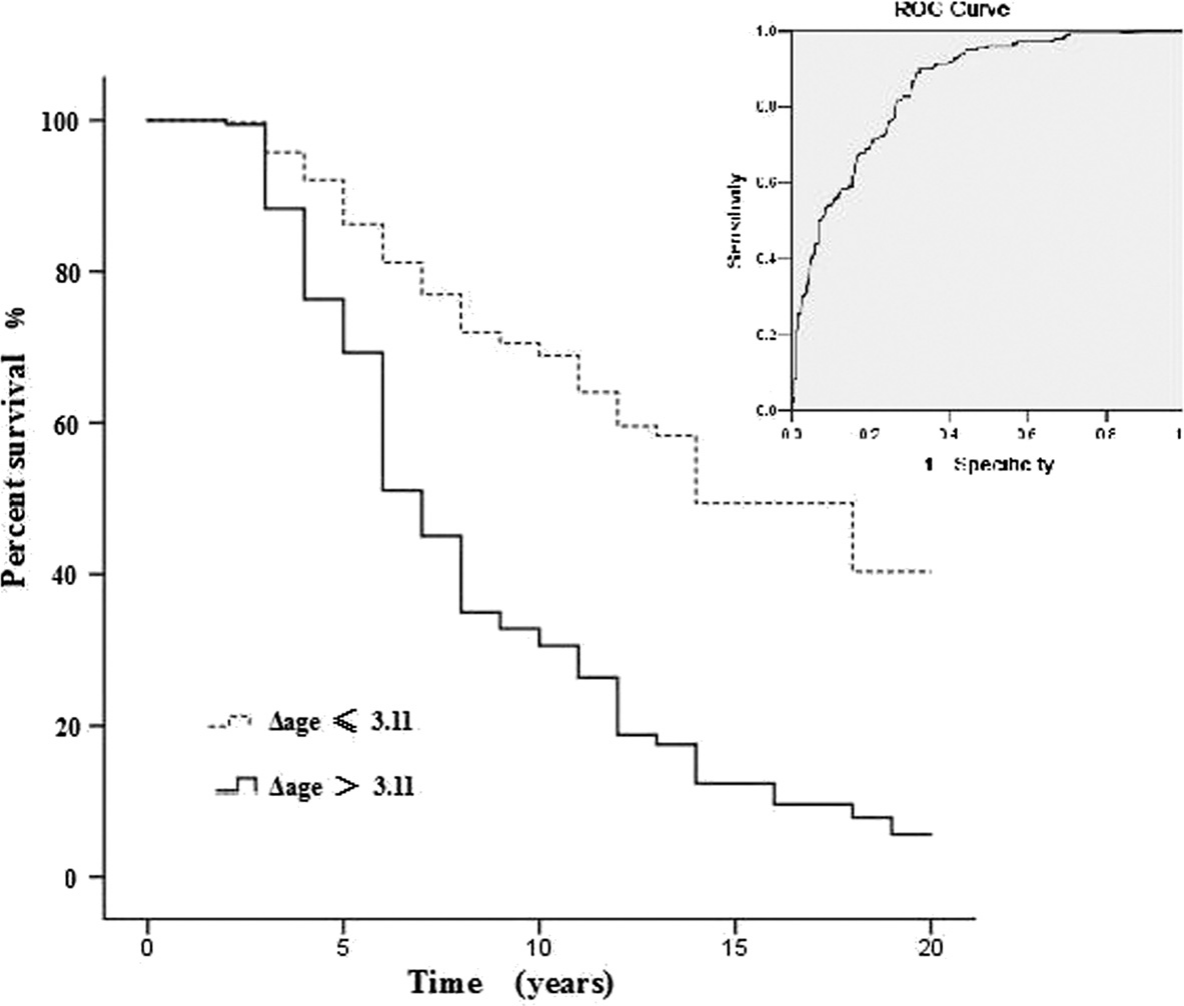
**ROC curve and Kaplan-Meier survival curve of the accuracy of Δage for prognostication (n = 1007).** The AUROC was 0.852 (95% CI 0.829–0.876), which indicates relatively good discrimination. It is analyzed using Kaplan-Meier survival model, including 649 cases with Δage ≤3.11, and 358 cases with Δage > 3.11. The result revealed that higher Δage (>3.11) was associated with poor prognosis. The Chi-square test indicated that there was significant difference between the two groups (P < 0.01).

Using the Simple Model of Stomach Age, we obtained a new variable (Δage) for all participants. We used the pathological diagnosis as the gold standard, and divided the 1,007 subjects into two groups: the poor prognosis group (Group C) or better prognosis group (Groups A and B on histology). Then, we analyzed the model by the ROC curve and calculated the AUROC. The model showed relatively good discrimination, with an AUROC of 0.852 (95% CI, 0.829–0.876; Figure 6).

The sensitivity and specificity were calculated using various cut-off points ranging from 0.0 to 1.0. We chose the Youden’s index cut-off point that corresponded to the maximal value as the new standard. A final probability cut-off score of 3.11 was used to predict those at a high risk of a poor prognosis (>3.11) or a low risk of a poor prognosis (≤3.11). The sensitivity and specificity were 82.7% and 71.8%, respectively.

Finally, we analyzed the effects of Δage on prognosis using the Kaplan-Meier Survival Model (Figure 6). The comparisons revealed that higher Δage (>3.11) was associated with poor prognosis (*P* <0.01). This results indicated that attention should be paid to those patients whose Δage > 3.11, due to the potential poor prognosis.

## Discussion

Aging is the progressive loss of metabolic and physiological functions, but its trajectory is not uniform, which suggests considerable variation exists in its biology [25]. This theory provides the basis of the concept of Stomach Age. People of the same biological age may have different Stomach Ages, and this variation may arise from a host of genetic and environmental factors. With the use of Stomach Age, we can differentiate between normal and abnormal stomach aging (the CAG in younger adults).

Telomere length in proliferative somatic cells is inversely correlated with age [26], and therefore the variation in Stomach Age recorded by FISH analysis of stomach biopsy samples could be an accurate means of describing the age of the stomach. According to our study, CAG patients have relatively shorter telomeres than normal and the Stomach Age of these patients is greater than their real age, which indicates that CAG could be regarded as a form of stomach aging. Thus, CAG in younger adults could be considered as premature aging of the stomach, which is a pathological process, and the mechanism of CAG in younger adults is quite different from that in more elderly people.

Further evidence to support this view is that the Δψm in CAG patients was significantly decreased, especially in younger adults. After exposure to the oxidative damage that is thought to contribute to aging, mitochondria are disproportionately damaged or destroyed and cannot maintain their Δψm [27]. This phenomena exists not only in many degenerative diseases (e.g., Parkinson’s disease) [28], but also in aging cells [29]. Our finding that the presence of CAG increased the decline of Δψm, especially in younger adults (*P* < 0.05); this indicates that CAG may accelerate aging in the stomach, leading to a greater Stomach Age. From these results, we hypothesize that Δage (Stomach Age - real age) could predict the severity of CAG and a higher Δage indicates abnormal aging of the stomach.

Moreover, age has been established as an independent risk factor for the development of gastric cancer in CAG patients, and a recent large cohort study showed that the age at the initial diagnosis was associated with the progression to more advanced lesions and to gastric cancer [30]. We hypothesize that those with abnormal biological stomach aging would have an increased risk of gastric cancer than those with a normal Stomach Age.

Although Stomach Age can be determined by FISH, the use of this method in large populations is unrealistic due to its high cost. For this reason, we modified the equation by logistic regression to obtain a simple form for clinical use. The questionnaire we used included numerous factors related to gastritis, which has been validated in previous studies [31-34]. To summary, firstly,we focused on eight factors that affect Stomach Age by using logistic regression (Table 3). There was no doubt that the endoscopy results influenced the model. Atrophy and IM can increase Stomach Age, whereas inflammation and its activity had no effect. Studies have shown that CAG is likely to advance gradually with increasing age, even in asymptomatic subjects [35] and there was an upward age-related trend of the prevalence of IM with increasing age [36], which is consistent with our results. Secondly, a family history of gastric cancer may also increase Stomach Age. The relationship between the family history of gastric cancer and the risk of CAG is controversial [37,38]. Our results support the idea that a family history of gastric cancer can accelerate aging of the stomach, which may lead to increased CAG. Thirdly, it is interesting that, in people with cardiovascular disease, aging of the stomach is accelerated, whereas the presence of gastroesophageal reflux disease (GERD) seems to have the reverse effect. This might be the result of hyperhomocysteinemia [39]. In addition, certain medications for the treatment of cardiovascular disease, such as aspirin, can cause gastric damage both through topical irritant effects on the epithelium and by systemic effects related to the suppression of mucosal prostaglandin synthesis [40], and thus may also lead to aging of the stomach. Aging is reported to be correlated with esophageal motor abnormalities such as GERD [41], but our findings are not the same. We hypothesize that our model for the determination of Stomach Age is more focused on secretion than on motor ability, as it is based on FISH measurements of telomere lengths, which are greater in normal epithelial cells than atrophic tissues. The mucosa and secretory glands are relatively complete in GERD; whether this has any effect on the measured Stomach Age should be investigated in future studies. Finally, junk food may also play an important role in Stomach Age. A recent study reported that trans fats, which are common in fast food, can accelerate brain aging [42]. We hypothesize that foods containing synthetic additives, such as preservatives, or those with a high fat content could also increase the Stomach Age.

Our study did not find any significant relationship between *H. pylori* infection and aging, which differs from the results of other studies [43,44]. There are at least two possible reasons. Firstly, because most of our patient information came from a cross-sectional study, *H. pylori* infection could have been present once but have been eliminated at the time of the biopsies. Another explanation is that the gastric environment no longer suited the growth conditions of *H. pylori*, and the bacteria could not survive in conditions with extensive atrophy [45,46].

In order to evaluate validity of the Simple Model of Stomach Age, we compared it with the results of FISH intensity by paired-*t* test which indicated no difference between them. The ability to predict disease’s prognosis of Simple Model was evaluated by ROC curve with large sample number, in which the cut-off point of Δage >3.11 is used to predict the high risk of getting poor prognosis (sensitivity and specificity of 82.7% and 71.8%, respectively). That means those patients with Δage >3.11 may develop to a worse prognosis in the future which should be closely follow-up and regularly examination of endoscopy should be provided to them.

However, the majority of CAG patients observed for a maximum of 16.5 years did not develop gastric cancer [30], which suggested that a surveillance program for all CAG patients may not be cost-effective. Our results showed that a subset of patients at a higher risk for GC could be easily identified by using the cut-off of Δage >3.11 at the time of the CAG diagnosis, which potentially enables these patients (Δage >3.11) to be selected for warranted endoscopy surveillance without unnecessarily increasing the healthcare costs in this field.

## Conclusion

In conclusion, this study provides a definition of Stomach Age, and demonstrates that people of the same true age may have different Stomach Ages. Attention should be paid to those with a greater Stomach Age than expected, especially in younger adults, as their mechanism of CAG is pathologically different to those in elderly patients, with a greater risk of developing gastric cancer over a lifetime. The Δage as calculated from the Simple Model was related to the prognosis for developing worsened CAG and the cut-off of >3.11 should be considered as a criterion to select a subgroup of CAG patients for gastric cancer surveillance.

## Competing interests

The authors declare that there is no conflict of interests.

## Authors’ contributions

Q-YG, Z-HW, YC (study concept and design; analysis and interpretation of data; drafting of the manuscript; critical revision of the manuscript for important intellectual content). J-QS, K-HZ, R-HS, J-MX, W-CC, X-LZ, S-DL, Y-XC (acquisition of data). Y-YS (statistical analysis). J-YF (study concept and design; study supervision). All authors read and approved the final manuscript.

## Pre-publication history

The pre-publication history for this paper can be accessed here:

http://www.biomedcentral.com/1472-6890/14/29/prepub
